# Effect of storage levels of nitric oxide derivatives in blood components

**DOI:** 10.12688/f1000research.1-35.v1

**Published:** 2012-10-22

**Authors:** Melissa A Qazi, Fabiola Rizzatti, Barbora Piknova, Nathawut Sibmooh, David F Stroncek, Alan N Schechter

**Affiliations:** 1 Molecular Medicine Branch, National Institutes of Diabetes and Digestive and Kidney Diseases, National Institutes of Health, Bethesda, MD 20892, USA; 2 Federal University of São Carlos, São Paulo, Brazil; 3 Department of Pharmacology, Faculty of Science, Mahidol University, Rama 6 Rd., Payathai Rajathewee, Bangkok 10400, Thailand, Thailand; 4 Department of Transfusion Medicine, National Institutes of Health, Bethesda, MD 220892, USA

## Abstract

**Background:** Potential deleterious effects of red blood cell (RBC) transfusions, especially from blood kept at length, have been ascribed to biochemical changes during storage, including those of nitric oxide (NO) metabolism.

**Study methods and design:** In this study, NO metabolites, nitrite and nitrate, were quantified in RBCs and whole blood with time of storage. Whole blood (WB), leukoreduced (LR), and non-leukoreduced (NLR) components were obtained from healthy volunteer donors and stored in polyvinyl chloride bags for 42 days. Nitrite and nitrate were measured using reductive gas-phase chemiluminescence.

**Results:** Nitrite concentrations initially decreased rapidly from about 150nmol/L, but stabilized at about 44nmol/L in room air for up to 42 days. Nitrate concentrations remained stable during storage at about 35µmol/L. Cells from bags maintained in an argon chamber showed decreased nitrite levels compared to those maintained in room air. Inhibition of enzymes implicated in the NO cycle did not alter nitrite levels.

**Conclusion:** As erythrocytes may contribute to the control of blood flow and oxygen delivery through reduction of nitrite to NO under hypoxic conditions, the present findings provide insight into possible effects of blood transfusion. These measurements may explain some adverse effects of RBC transfusion and suggest ways of optimizing the preservation of stored blood.

## Introduction

The field of transfusion medicine has experienced much controversy surrounding the safety and efficacy of current transfusion practices. Potentially deleterious effects that are seen with blood storage–including, but not limited to, declines in 2,3-DPG and ATP, as well as increases in potassium content and in free hemoglobin and iron (due to hemolysis of red cells)–have been termed the “storage lesion” ^1, 2^. Our limited understanding of the significance of this storage lesion or storage-induced physiological changes is at the root of a current debate about the efficacy of using long-stored blood. Reports of post-transfusion complications, such as multiple organ failure, sepsis, and even small general increases in morbidity and mortality, have fueled much concern ^1, 2^, but clinical investigation has yielded conflicting opinions about the actual impact of the age of blood on transfusion outcomes. Some studies suggest that transfusion with stored blood results in greater post-operative complications than transfusions with fresh blood because of the deleterious effects of storage ^3– 5^. However, several other studies have presented results that indicate no definitive difference between transfusion outcomes with fresh blood or aged blood ^6– 8^, or if there is an effect, it is probably small ^9, 10^. Infusing stored blood with augmented 2,3-DPG and ATP has resulted in improved transfusion outcomes ^11^, and a similar approach is now being considered to target other potentially deleterious biochemical changes.

Among the observed adverse effects of blood transfusions, reduced oxygen delivery and reduced vasodilatory capabilities of stored RBCs are considered especially critical factors ^1, 2, 12^. It is now known that one of the primary vasodilators and regulators of blood flow is the endothelium-derived relaxing factor, nitric oxide (NO) ^13^. Substantial production of NO occurs within tissues via several mechanisms. Initially, conversion of L-arginine to NO was thought to be primarily via endothelial nitric oxide synthase (eNOS) ^14– 16^ and to a lesser extent, via neuronal nitric oxide synthase (nNOS) and inducible nitric oxide synthase (iNOS) enzymes ^17^. It has recently been realized that, in addition to NOS synthesis, nitrite reduction to NO may be catalyzed by the enzymatic action of xanthine oxidoreductase, nonenzymatic disproportionation, and reduction by deoxyhemoglobin in blood and by other heme-proteins in various tissues ^18– 23^. Indeed, nitrite ions may be the major storage pool of NO bioactivity. On the other hand, erythrocytic hemoglobin is a major sink for the destruction of NO, and cell-free hemoglobin is an even more effective sink for NO ^24^. Clearly the physiological and potentially pathological effects of red cell transfusions will be affected by any changes in these NO synthetic and destructive processes prior to, during, and immediately after red cell administration.

Currently, there is interest in the investigation of potential clinical consequences of changes in NO derivatives during storage, especially with respect to oxygen delivery and vasodilatory capabilities of transfused blood, as well as any association with transfusion-related complications. Two approaches to this have surfaced from our understanding of the metabolism of NO. In one, the nitrite/NO pathway is implicated-nitrite is a major storage pool of NO that can interconvert directly or indirectly with NO. In fact, studies on platelets have elucidated a functional role of nitrite as a modulator of platelet aggregation under hypoxic conditions ^25, 26^. In another approach, S-nitrosylated hemoglobin (SNOHb) is implicated-indeed, it was proposed that the amount of SNOHb is responsible for the quality of stored red cells, and that replenishing SNOHb would be therapeutically helpful, supposedly restoring the oxygen-transport and vasodilatory capabilities of RBCs. However, this theory has been questioned on several grounds ^20, 27– 29^. Evaluation of NO availability with respect to hemoglobin-mediated reductive mechanisms thus appears warranted.

In the present study, we quantified the main nitric oxide metabolites–nitrite and nitrate–as a function of duration of blood storage. We investigated blood stored under standard blood bank protocols, as well as blood stored in an argon chamber, to prevent gas exchange with the surrounding air. We also addressed the role of enzyme inhibition in NO metabolite composition in stored blood. Our results demonstrated a stable nitrate concentration throughout storage but a gradual decline in nitrite concentration after the initial rapid destruction upon venisection. Stored blood consistently maintained low levels of nitrite until the end of storage. The potential implications of these results for blood transfusion therapies are discussed.

## Materials and methods

### Human blood donors and study design

Ten healthy volunteer donors enrolled in an Institutional Review Board approved protocol each provided one 450mL unit of blood for this study. Blood was drawn using the standard phlebotomy method and stored in polyvinyl chloride (PVC) bags for up to 42 days at 4°C, per standard blood bank protocol. An additional 20mL of whole blood was collected in 10.0mL Becton Dickinson (BD) Vacutainer ^®^s for immediate processing. Each single unit of blood was split into units of WB, NLR, and LR blood components. Six units were used to measure changes in WB, LR, and NLR blood samples over 42 days. Three of these units were randomly designated for room air storage and the other three were for argon chamber storage. Supernatants from these six units were used to measure their respective nitrite and nitrate values when stored in either room air or the argon chamber, and small amounts of blood from each bag were used to measure the pO _2_ levels as well. The four remaining units were used for the enzyme inhibition studies, with 2 units for L-NAME and 2 units for acetazolamide. Each unit was split into LR and NLR control units and LR and NLR units for enzyme inhibitor administration.

To test immediate nitrite decay, a separate pool of donors was used.

### Separation of blood components

**Supplemental Figure 1.**
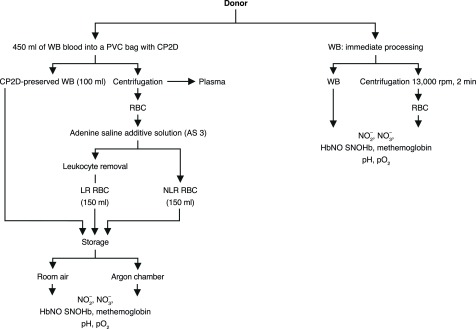
Outline of study design.

Whole blood collected using a citrate phosphate double dextrose anticoagulant (CP2D) PVC bag was split three ways–whole blood (WB), non-leukoreduced (NLR) blood component, and leukoreduced (LR) blood component (see Supplemental Figure 1 for an overview of the protocol). 100mL of WB was drawn into a new PVC bag. The remaining blood in the original CP2D unit was centrifuged and the plasma was removed; these RBCs were mixed with adenine-saline (AS-3) solution and comprised the NLR component. 150mL of this unit were filtered through an RCM1 Leukocyte Filter (Pall Corp., East Hills, NY) to obtain the LR component. All components were stored in identical AS-3 PVC bags. Blood separation occurred at 20 to 24°C and required approximately 1 hour.

### Blood storage

Blood components were either stored in room air for 42 days at 4°C - standard blood bank storage conditions, or in an air-tight chamber (Fisher Scientific, Vacuum Dessicator Cabinet, Suwanee, GA) for 42 days at 4°C in an argon atmosphere (Roberts Oxygen Co., 99% pure argon, Rockville, MD).

### Sample preparation and testing

Samples were collected for analysis of nitrite, nitrate, SNOHb, iron nitrosyl hemoglobin (HbNO), and methemoglobin (MetHb) in all components. Immediately upon venisection, baseline samples of heparinized WB and RBC were taken. Aliquots of blood were thereafter drawn from storage bags using a liquid transfer set (Charter Medical, Winston-Salem, NC) to maintain sterile conditions. Three replicates of each sample were collected in Eppendorf tubes everyday for the first seven days of storage, and every 2–3 days following that, until day 42. Upon collection, samples were placed on dry ice and maintained in a -80°C freezer until thawed on regular ice prior to chemiluminescent analysis. An Abbott i-STAT cartridge reader (Abbott Laboratories Inc., Portable Clinical Analyzer, East Windsor, NJ) was used to determine pH and pO _2_ levels (using CG8 ^+^ and G3 ^+^ cartridges) at the time of sample collection.

### Blood nitrite and nitrate

A standard protocol was used to determine NO _2_^-^ and NO _3_^-^ levels in the three blood components ^30, 31^. “Stop solution” (K _3_Fe(CN) _6_, N-ethylmaleimide, water, NP40) was added to blood to maintain nitrite levels until sample analysis ^21^. A 1:4 dilution of “stop solution” to blood was vortexed and placed on dry ice. At the time of sample analysis, a 1:1 dilution of 99.9% pure methanol and thawed sample was centrifuged for 2min at 13,000rpm; the supernatant was immediately injected into the chemiluminescent nitric oxide analyzer (NOA, Sievers, Model 280 NO analyzer, Boulder, CO) using helium as the carrier gas. The triiodide (I _3_^-^) ozone-based chemiluminescent assay was used to analyze nitrite levels. To analyze nitrate, deionized water (Millipore CQ-Gard, Bedford, MA) was added to blood to lyse cells. A 9:1 dilution of deionized water to blood was vortexed and placed on dry ice. At the time of sample analysis, a 3:1 dilution of pure HPLC grade ethanol and thawed sample was centrifuged, and the supernatant was immediately analyzed using the Vanadium(III)chloride chemiluminescent assay. The VCl _3_ reaction solution was maintained at 90°C with helium as the carrier gas. 1µM nitrite and nitrate solutions were used to generate standard curves for comparisons and adjustments of sample nitrite and nitrate concentrations.

### SNOHb/HbNO

A standard protocol was used to determine SNOHb and HbNO levels in all components ^30, 31^. A thiol-stabilization solution (NEM-DPTA; K _3_Fe(CN) _6_, N-ethylmaleimide, Diethylenetriaminepenta acetic acid, NP40, water) was added to blood to maintain SNOHb and HbNO levels by inhibiting additional thiol reactions. A 4:1 dilution of NEM-DPTA to blood was vortexed and placed on dry ice. A 9:1 dilution of sample and 5% acid sulfanilamide (AS) was incubated for 5min; half was injected into the NOA (I _3_^-^ assay) to give combined SNOHb and HbNO levels. The remaining sample was incubated with 50mM HgCl _2_, then incubated again with 5% AS, and injected into the NOA to give HbNO levels.

### Supernatants and saline

Blood components were centrifuged at 13,000rpm for 5min and WB, NLR, and LR supernatants were collected. Aliquots of saline were collected directly from control PVC bags using a liquid transfer set. The aforementioned I _3_^-^ and VCl _3_ assays were used for WB, NLR, and LR supernatants and saline samples. As samples were already separated from the blood pellet, treatment with methanol and ethanol was unnecessary.

### Methemoglobin

Methemoglobin (MetHb) analysis was performed at each sample collection. Pre-storage and storage values (up to 42 days) were evaluated using a CO-Oximeter (Radiometer America Inc., OSM3 Hemoxymeter, Cleveland, OH).

### Nitrite decay

Fresh whole blood collected in heparinized Vacutainer ^®^s was immediately sampled for pre-storage nitrite and nitrate levels using the aforementioned protocols. The first 5 hours of ex vivo NO _2_^-^ decay was studied for WB and RBCs only. Aliquots were collected at time = 0, 5, 10, 15, 20, 30, 60, 120, 180, 240, and 300 (in minutes), where time points do not account for a 3–5 minute delay in receiving samples from the phlebotomist. 1mL of whole blood was centrifuged at 13,000rpm for 2min at each interval to obtain RBCs.

### Enzyme inhibition

Bags of LR and NLR RBCs were stored for 7 days and infused once daily with one of three inhibitors: N-Nitro-L-arginine methyl ester hydrochloride (L-NAME, PBS; final concentration: 1mM, Sigma-Aldrich Co.) ^32^ for NOS inhibition, acetazolamide (acetazolamide, DMSO; final concentration: 100µM, Sigma-Aldrich Co.) ^33^ for carbonic anhydrase inhibition, and oxypurinol (oxypurinol, NaOH, Tris-Ringer buffer; final concentration: 0.1mM, Sigma-Aldrich Co.) ^34^ for xanthine oxidase inhibition. All inhibitors were administered using a liquid transfer set. Controls were infused once daily with identical saline volume. Blood and supernatant samples were prepared 1 hour after infusion and tested per the aforementioned methods for nitrite analysis.

### Data analysis

Data were recorded using the NOAnalysis 3.21 Liquid software. OriginLab 7 was used to analyze data and calculate the amount of NO detected, by evaluating the area under the peaks and comparing them to known standards. Nitrite and nitrate values were corrected by subtracting the amount of nitrite present in methanol and stop solution, and the amount of nitrate in ethanol and water, respectively. SNOHb was determined by subtracting HbNO levels from cumulative HbNO and SNOHb levels.

### Statistical analysis

*GraphPad Prism 4* was used for statistical analysis and graphical representation of data (mean ± SEM). A one-way ANOVA test with the Bonferroni multiple comparison analysis was used to determine statistical significance. Results with a p-value of less than 0.05 were considered significant.

## Results

### Changes in nitrite levels over the duration of storage

**Figure 1.**
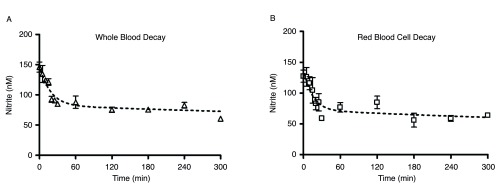
Whole blood (1A) and red blood cell (1B) nitrite decay over the first 5 hours following blood draw. Blood components were kept in room air at 24°C; number of donors, n=6 ( **A**), n=4 ( **B**). Time points above do not account for a 3–5 minute delay in receipt of blood from phlebotomist.

Nitrite levels showed the expected very rapid decay in both whole blood and red blood cells in the hours immediately following venisection ( Figure 1). Blood was kept in air at room temperature for the duration of these measurements. At t = 0, the nitrite concentration in whole blood ( Figure 1A) was about 150nM; by 60min, endogenous blood nitrite levels had decreased to about 85nM, and by 5 hours, nitrite levels had decreased to about 65nM. The values for the first 60 minutes are consistent with previously reported data ^21^. Red blood cell preparations, in which the first measurements were delayed by the processing time (approximately 3–5 minutes after receipt from the phlebotomist), demonstrated comparable behavior ( Figure 1B), but the higher initial values were lost during this time.

Changes in whole blood and red blood cell nitrite levels over the duration of storage. Whole blood (1A) and red blood cell (1B) nitrite decay over the first 5 hours following blood draw. Blood components were kept in room air at 24°C; number of donors, n=6 (A), n=4 (B). Time points above do not account for a 3-5 minute delay in receipt of blood from phlebotomist. To view the data behind the graphs, access 'show all items' above.

**Figure 2.**
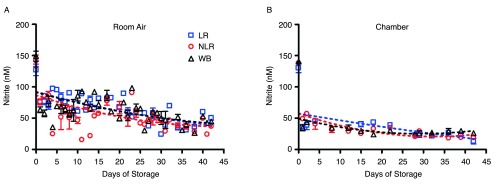
Time-dependent changes in nitrite concentration during storage. Blood components stored in the three forms noted were kept for 42 days at 4°C in either room air ( **2A**) or an argon chamber ( **2B**), to emulate aerobic and hypoxic conditions, respectively; number of donors, n=3 ( **A**), n=3 ( **B**).

**Supplemental Figure 2.**
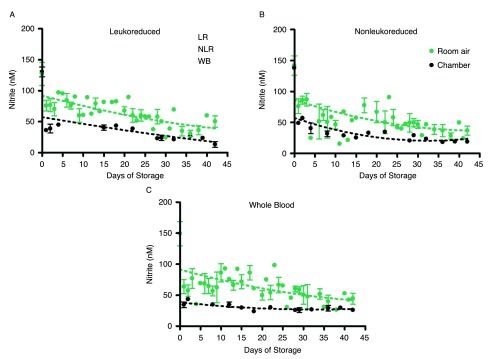
Time-dependent room air and chamber changes in nitrite concentration for individual stored blood components; number of donors, n=3 (room air), n=3 (chamber).

**Figure 3.**
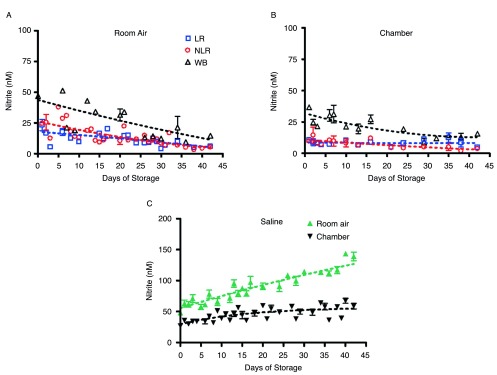
Nitrite concentration in supernatants and saline stored in room air or an argon chamber. Figure 3A shows the nitrite concentration in supernatants stored in room air, number of donors, n=3, while Figure 3B shows the same for supernatants stored in an argon chamber, number of donors, n=3. Nitrite concentrations in saline controls stored under both conditions are shown in Figure 3C, number of donors, n=6 (room air n=3, argon chamber n=3).

Nitrite underwent additional, but slower decay in all three product forms and their supernatants over 42 days of storage, but the nitrite concentration leveled off in room air samples at about 44nM ( Figure 2A) in all three blood components. This gradual decrease in concentration and leveling was found in both air and argon chamber stored blood ( Figure 2B). However, comparison of blood nitrite levels from air (Figure 2A) and the argon chamber ( Figure 2B) reveals a noticeable depression in nitrite values in the chamber environment that is consistent throughout the storage period. RBCs stored in room air had nitrite concentrations of 42 ± 4nM on day 42; while the argon chamber samples decreased in concentration to 16 ± 3nM on day 42. WB stored in room air reached nitrite concentrations of 44 ± 8nM, while WB stored in the chamber reached nitrite concentrations of 26 ± 3nM by the end of the storage period (p>0.05). Under both storage conditions, nitrite levels were very similar (within the error of this assay) for the three types of cell preparations–whole blood, non-leukoreduced RBCs, and leukoreduced RBCs-for the duration of the experiment ( Supplemental Figure 2). However, the higher values in room air as compared to chamber-stored samples suggest additional factors affecting production and/or destruction of nitrite ions. Supplemental Figure 2 presents curve-fitting of these data, displaying room air and chamber nitrite decay for the individual blood components. The same trends were seen in the nitrite concentrations of supernatants for each of the three blood components ( Figures 3A and 3B). Nitrite concentration in supernatants was significantly lower than that in blood components, confirming nitrite localization in erythrocytes and the findings of previous studies ^21^.

Changes in the three blood forms nitrite levels over the duration of storage. Figure 2. Time-dependent changes in nitrite concentration during storage. Blood components stored in the three forms noted were kept for 42 days at 4°C in either room air (2A) or an argon chamber (2B), to emulate aerobic and hypoxic conditions, respectively; number of donors, n=3 (A), n=3 (B). Supplemental Figure 2. Time-dependent room air and chamber changes in nitrite concentration for individual stored blood components; number of donors, n=3 (room air), n=3 (chamber). To view the data behind the graphs, access 'show all items' above.

Nitrite concentration in supernatants and saline stored in room air and argon chamber. Nitrite concentration in supernatants and saline stored in room air or an argon chamber. Fig. 3A shows the nitrite concentration in supernatants stored in room air, number of donors, n=3, while Fig. 3B shows the same for supernatants stored in an argon chamber, number of donors, n=3. Nitrite concentrations in saline controls stored under both conditions are shown in Fig. 3C, number of donors, n=6 (room air n=3, argon chamber n=3). To view the data behind the graph, access 'show all items' above.

In saline stored in PVC bags, nitrite levels varied greatly based on storage conditions ( Figure 3C). Saline stored in room air experienced a gradual increase in nitrite concentration from 62 ± 6nM on day 1 to 140 ± 7nM on day 42. Saline stored in the argon chamber remained relatively steady over the storage period, as expected, showing a slight increase in nitrite concentration from 36 ± 2nM on day 1 to 58 ± 5nM on day 42.

### Nitrate remains constant for the duration of storage

**Figure 4.**
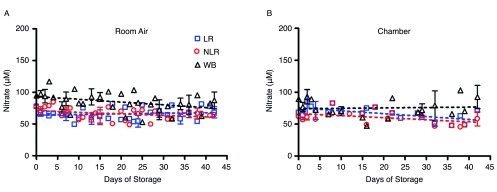
Effect of storage on nitrate concentration in blood stored for 42 days in room air (4A) or an argon chamber (4B); number of donors, n=3 (A), n=3 (B).

In contrast to the results with nitrite ions, nitrate levels in WB, in NLR and LR RBCs, and in supernatant samples remained steady for the duration of blood storage ( Figure 4). Whole blood nitrate levels were slightly higher than either the non-leukoreduced or the leukoreduced RBC components stored either in air or in the argon chamber. In room air, WB nitrate concentration was about 47 ± 2µM, while NLR and LR nitrate concentrations were about 34 ± 2µM. For blood stored in the argon chamber, WB nitrate levels were lower than respective room air samples, with chamber nitrate remaining at about 37 ± 3µM. LR and NLR RBC nitrate levels in the chamber were unchanged when compared to LR and NLR RBCs stored in air, remaining at about 35 ± 2µM. Supernatant nitrate levels (measured after centrifugation of packed red cells or whole blood) followed the same trend as the blood samples, remaining steady for the duration of storage and exhibiting similar nitrate concentrations when stored in an argon chamber (data not shown).

Effect of storage on the three blood forms nitrate concentration Effect of storage on nitrate concentration in blood stored for 42 days in room air (4A) or an argon chamber (4B); number of donors, n=3 (A), n=3 (B). To view the data behind the graphs, access 'show all items' above.

### HbNO, SNOHb, and MetHb levels

**Figure 5.**
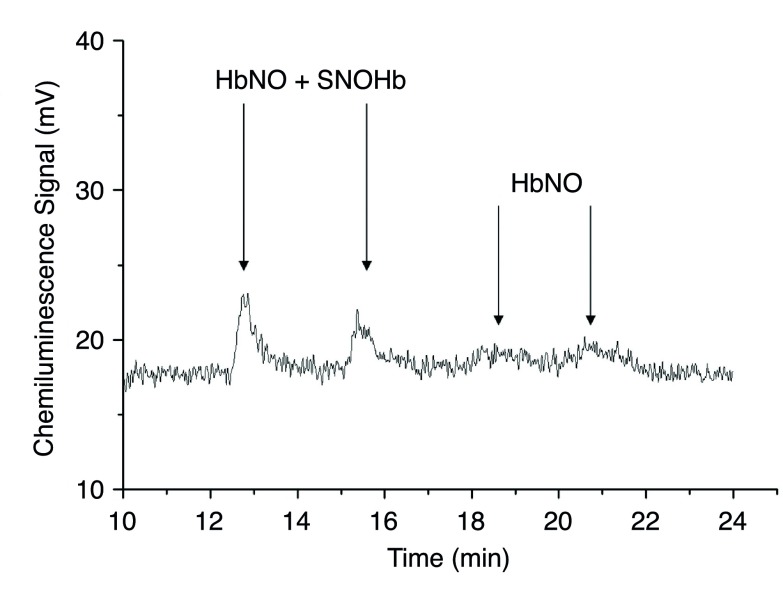
SNOHb levels in fresh blood (assay performed in the first hour after venisection). Gas-phase chemiluminescence signals used to determine SNOHb concentration. The peaks from two samples in the first 20 minutes of storage are shown; SNOHb concentration is ascertained by subtracting the HbNO peak from the composite of SNOHb plus HbNO after treatment with HgCl _2_ and acid sulfanilamide. The values of SNOHb, near the sensitivity of the method, are less than 30nM, while HbNO is barely detectable. Neither peak was detected after 1hr of storage.

The gas-phase chemiluminescence I _3_^-^ assay was used to determine SNOHb and HbNO levels in blood components stored in both room air and chamber conditions. Figure 5 shows HbNO and SNOHb levels as detected by the NOA at two time points in the first hour following venisection. However, levels of HbNO and SNOHb were virtually undetectable 1 hour after blood collection and in stored blood thereafter.

**Figure 6.**
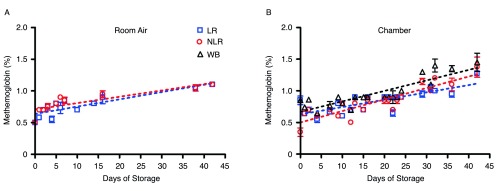
Change in MetHb levels in room air (6A) and chamber (6B) samples over 42 days of storage; number of donors, n=2 (A), n=2 (B).

A CO-oximeter was used to measure MetHb levels in all three blood components stored in both room air and chamber conditions. Figure 6 shows the gradual increase in MetHb from nearly 0.5% to just above 1% during the storage period. MetHb concentration is expressed as percentage of total hemoglobin concentration.

HbNO and SNOHb levels SNOHb levels in fresh blood (assay performed in the first hour after venisection). Gas-phase chemiluminescence signals used to determine SNOHb concentration. The peaks from two samples in the first 20 minutes of storage are shown; SNOHb concentration is ascertained by subtracting the HbNO peak from the composite of SNOHb plus HbNO after treatment with HgCl2 and acid sulfanilamide. The values of SNOHb, near the sensitivity of the method, are less than 30nM, while HbNO is barely detectable. Neither peak was detected after 1hr of storage. To view the data behind the graphs, access 'show all items' above.

MetHb levels in room air and argon chamber. Change in MetHb levels in room air (6A) and chamber (6B) samples over 42 days of storage; number of donors, n=2 (A), n=2 (B). To view the data behind the graphs, access 'show all items' above.

### Blood storage under standard conditions vs. hypoxic conditions

**Supplemental Figure 3.**
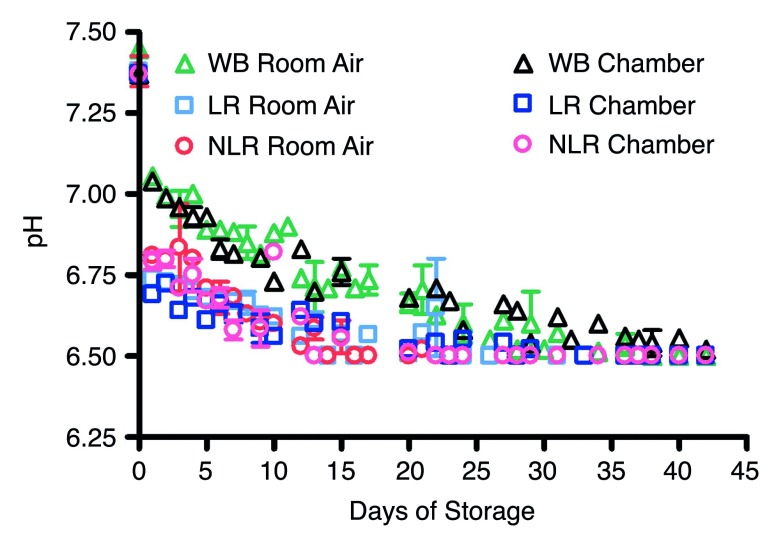
Room air and argon chamber comparisons of pH levels in stored blood; number of donors, n=3 (room air), n=3 (chamber). Note: i-STAT apparatus does not detect pH levels below 6.5; values read as <6.5 were plotted at 6.5.

Room air at 4°C satisfied standard conditions for blood storage under current American Association of Blood Banks (AABB) Transfusion Medicine protocols; the argon chamber prevented gas exchange with ambient air and also mimicked blood storage under hypoxic conditions. Blood components stored in room air demonstrated a gradual rise in partial pressure of oxygen over the first three weeks and leveled off thereafter. pO _2_ of blood components stored in the argon chamber remained at the levels of venous blood initially drawn or even showed a small decrease during storage. The pH of the samples stored in air was measured over the 42 days and gradually decreased from about 7.4 to below 6.5 ( Supplemental Figure 3). This is consistent with previous reports ^35^. The time in which pH levels fell below 6.5 differed among the blood products, with NLR RBC pH falling after the first 13–16 days, LR RBC after 23–24 days, and WB after about 38 days of storage.

Room air and argon chamber comparisons of pH levels in stored blood. Room air and argon chamber comparisons of pH levels in stored blood; number of donors, n=3 (room air), n=3 (chamber). Note: i-STAT apparatus does not detect pH levels below 6.5; values read as < 6.5 were plotted at 6.5. To view the data behind the graph, access 'show all items' above.

### Inhibition of NO-producing enzymes

**Supplemental Figure 4.**
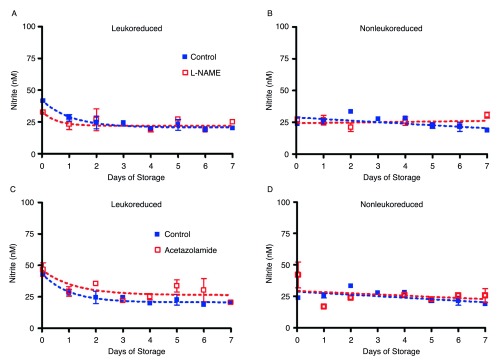
Enzyme inhibition of NO production. Inhibition of NOS with L-NAME in leukoreduced RBCs (S4A) and non-leukoreduced RBCs (S4B); number of donors, n=2. S4C and S4D show inhibition of carbonic anhydrase with acetazolamide in leukoreduced RBCs and in non-leukoreduced RBCs, respectively; number of donors, n=2. All bags were stored in room air at 4°C for 7 days.

It has been suggested that xanthine oxidase may take on the role of the main enzyme responsible for converting nitrite to vasoactive NO in hypoxia ^36^. In our study, however, several key NO-generating enzymes–NOS, carbonic anhydrase, and xanthine oxidase–were inhibited with L-NAME, acetazolamide, and oxypurinol, respectively. The amount of nitrite measured in the bags did not change as a result of NOS enzyme inhibition with L-NAME or carbonic anhydrase inhibition with acetazolamide ( Supplemental Figure 4) over seven days of storage as compared to control LR or NLR red cells. The data for oxypurinol inhibition of xanthine oxidase were similar (data not shown).

Inhibition of NO-producing enzymes Change in MetHb levels in room air (6A) and chamber (6B) samples over 42 days of storage; number of donors, n=2 (A), n=2 (B). To view the data behind the graphs, access 'show all items' above.

## Discussion

There is much concern, but also much controversy, about whether storage of red blood cells for transfusion decreases their therapeutic efficacy, or even leads to harm upon administration. The history of transfusion medicine has seen the development of technologies to allow increasingly extended storage of blood products, especially erythrocytes ^37^; in the last century, such advances have revolutionized medical practice by allowing blood to become a readily available therapeutic agent. In recent years, these advances have been called into question by the perception of possible deleterious effects of long-stored blood as compared to relatively new units of blood. Such negative effects, which are said to be associated with a “storage lesion”, have been ascribed to some of the many biochemical changes that occur upon storage, including depletion of 2,3-DPG, ATP, and potassium ions, or even cellular changes, including increased rigidity or hemolysis, with resultant increases in free hemoglobin and iron in the transfusion recipients ^1, 2, 37^. Although no significant hemolysis was seen in this study, a recent finding indicates that there may be some increase during the final week of storage ^38^.

Clearly, many of these storage-related red cell changes, which could affect blood flow and oxygen delivery in the recipients, reverse rapidly upon infusion, and probably account for the fact that survival of 75% of transfused cells at 24 hours can be used as an achievable criterion for efficacy following storage ^39^. (However, one might imagine that such *in vivo* repletion might not be rapid enough to avoid deleterious effects with very large volume transfusions). Indeed, the clinical evidence for blood age-associated transfusion complications is itself uncertain. It is clear that any such effects are relatively small compared to the efficacy of transfusions, which likely explains the difficulty of convincingly demonstrating them in small trials, where there are many confounding variables. However, in an era of therapy optimization, it is very important that we examine whether prolonged blood storage causes any changes, reversible or irreversible, that may interfere with the goals of transfusion medicine.

The importance of NO and the related oxides of nitrogen to determining blood flow and other biological phenomena has led to widespread focus on the role of NO in physiology and pathophysiology, and as of late, also during blood storage. It has long been recognized that both intraerythrocytic and free hemoglobin readily destroy NO. In a recently published reviews, a causal relationship between hemolysis-dependent changes in NO functionality and the storage lesion was presented, and the importance of contextual understanding of these changes through NO metabolites is addressed ^40, 41^. The recent recognition that red cells may transduce NO bioactivity by transporting NO in an endocrine-like fashion ^42^ has focused interest on measuring parameters related to these processes in stored red cells, as they may balance the destructive mechanisms. Much recent work has focused on the ability of deoxyhemoglobin and other red cell-related proteins, xanthine oxidoreductase in particular ^43^, to reduce red cell or plasma nitrite ions to NO. A recent study of NO-related metabolic changes during platelet storage also suggests the possibility of platelet consumption of nitrite ^26^. An older hypothesis, in which NO forms S-nitrosohemoglobin (SNOHb) upon reaction with oxyhemoglobin but then dissociates upon deoxygenation, has some adherents, although recent experiments involving transgenic animal models seem to largely negate this theory ^44^.

Nitrite in red cells and plasma may be considered as the primary storage form of NO because of its relative abundance in blood and increasing evidence of its physiological and pharmacological importance. Nitrite and nitrate ions are the major oxidation products of NO metabolism and are produced in the body by reaction with oxygen, hemoglobin, ceruloplasmin and possibly other molecules ^45^. In addition, nitrite and nitrate are ingested with food; nitrite can also be produced from nitrate by salivary bacteria ^46^. Measurement of nitrite levels in blood have been suggested as an index of NO bioavailability for epidemiological studies of cardiovascular disease severity ^47^.

Our approach to the question of NO bioavailability in stored red cells has been to measure nitrite and nitrate as a function of duration and conditions of storage as occurs in transfusion practice. Our major finding is that, although much red cell nitrite is rapidly lost during the first few hours of storage, nitrite levels continue to deplete, albeit at a much slower rate, to a clear plateau of about 44nM in the last two weeks of storage. This phenomenon is almost identical in red cells stored with or without leukodepletion and those stored as whole blood; this suggests that interactions with white blood cells and platelets do not significantly impact nitrite metabolic processes. However, while storage of blood in a closed chamber under argon did not change the progression of the nitrite depletion, this environment did lower overall nitrite values in a nearly uniform fashion, to a final concentration of about 20nM.

Red cell nitrate levels remained virtually unchanged from day 1 to day 42, at around 35µM (approximately 1000X the final concentration of nitrite), and were identical for red cells stored as leukoreduced, non-leukoreduced, and whole blood. The storage conditions, whether room air or argon chamber, did not affect these measurements. As expected, metHb concentrations under all experimental conditions remained around 1%, as previously shown ^34^. Levels of SNOHb during the first hour of analysis were barely detectable by gas-phase chemiluminescence after triiodide treatment, and were consequently unquantifiable.

Our nitrite results, which showed that nitrite levels stabilized and remained stable at nonzero values, were surprising. From previous studies of the reaction of hemoglobin with nitrite *in vitro*, we would predict that nitrite would largely disappear over the course of storage. Although we cannot offer a complete explanation for the mechanism of nitrite retention in the red cell, our investigation of this effect has led to several conclusions. First, we addressed the potential role of several enzymes implicated in NO cycle–NOS, xanthine oxidase, and carbonic anhydrase. Nitrite levels did not change upon inhibition of these enzymes, indicating that they are not likely contributors to the maintenance of the low nitrite concentrations. Second, HbNO is also an unlikely source of nitrite, as it is nearly unquantifiable after twenty minutes, with a half-life of ~79 minutes ^23^. Third, reduction of nitrate to nitrite also seems unlikely, since mammalian processes to account for such a reaction have not been described; however, the fact that nitrate remains steady does not necessarily exclude this process, as only a one-tenth of 1% change in nitrate could account for the maintenance of these nitrite levels. Finally, while bacteria are known to synthesize nitrite, bacterial contamination may be ruled out because standard blood storage protocol virtually eliminates the risk of such contamination. On the other hand, several explanations may account for at least part of this nitrite retention. Greater depletion of nitrite found in the argon chamber samples raises the possibility that nitrogen reactive species may enter the stored blood via diffusion from the atmosphere through the gas-permeable PVC bags, as confirmed by our saline solutions. Atmospheric nitrogen reactive species may therefore account for some of the nitrite that remains. Other potential mechanisms to explain nonzero values of nitrite at 42 days include the possibility of nitrite binding to RBC proteins (such as band 3 proteins or other members of the cytoskeletal protein scaffold via ionic interactions that could effectively shield nitrite from reacting with hemoglobin), hemoglobin–nitrite ionic interactions, and formation of nitrite-methemoglobin complexes.

NO and its metabolic products are likely to play an important role in maintenance of banked blood, as effective agents for transfusion therapies. Nitrite seems to be a source of great NO bioactivity that remains present in the blood for the duration of storage. Nitrate is also present in relative abundance to nitrite throughout storage. The presence of these metabolites in red cells up to 42 days of storage provides the possibility that maintenance of NO/nitrite levels and NO bioactivity is likely to occur via the nitrate-nitrite-NO pathway.
