# Supplementary figures and images for: Effect of storage levels of nitric oxide derivatives in blood components
Source: F1000Res. 2012 Oct 22;1:35. [Version 1] doi: 10.12688/f1000research.1-35.v1 (PMC3814924; doi:10.12688/f1000research.1-35.v1)

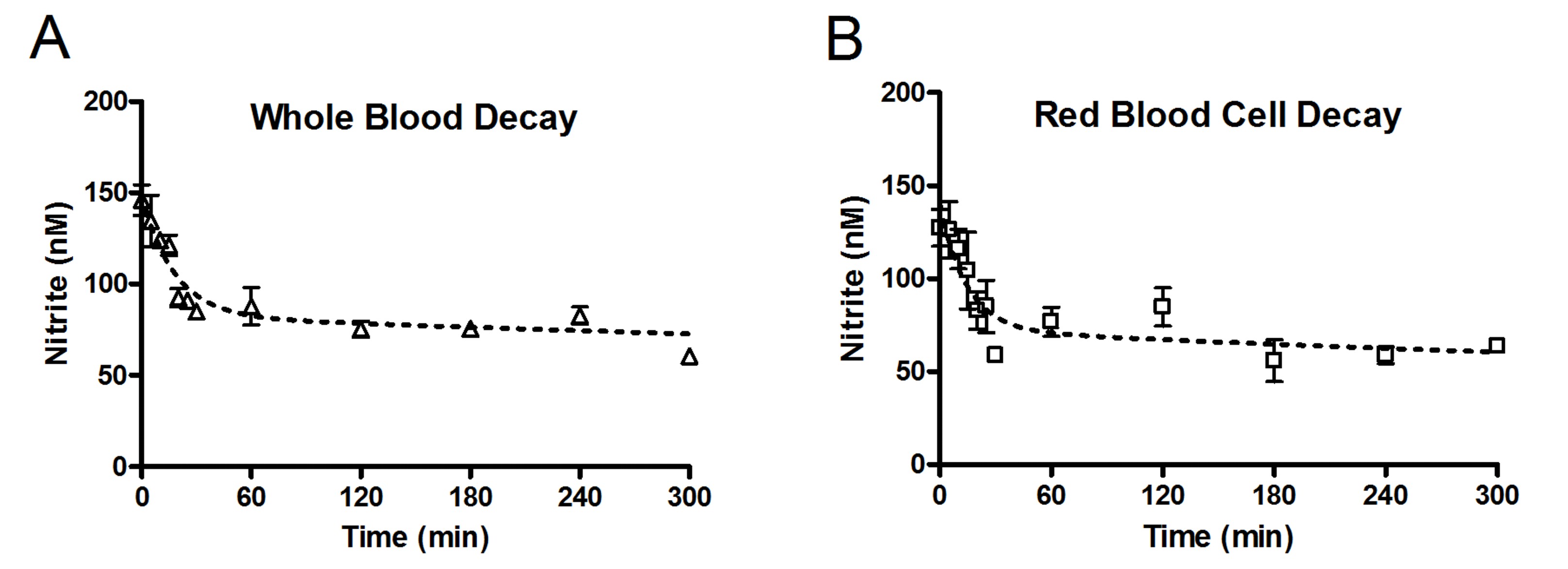

Supplement: Changes in whole blood and red blood cell nitrite levels over the duration of storage. — Whole blood (1A) and red blood cell (1B) nitrite decay over the first 5 hours following blood draw. Blood components were kept in room air at 24°C; number of donors, n=6 (A), n=4 (B). Time points above do not account for a 3-5 minute delay in receipt of blood from phlebotomist. To view the data behind the graphs, access 'show all items' above. [file f1000research-1-212-s0007.tgz › figure1.jpg]

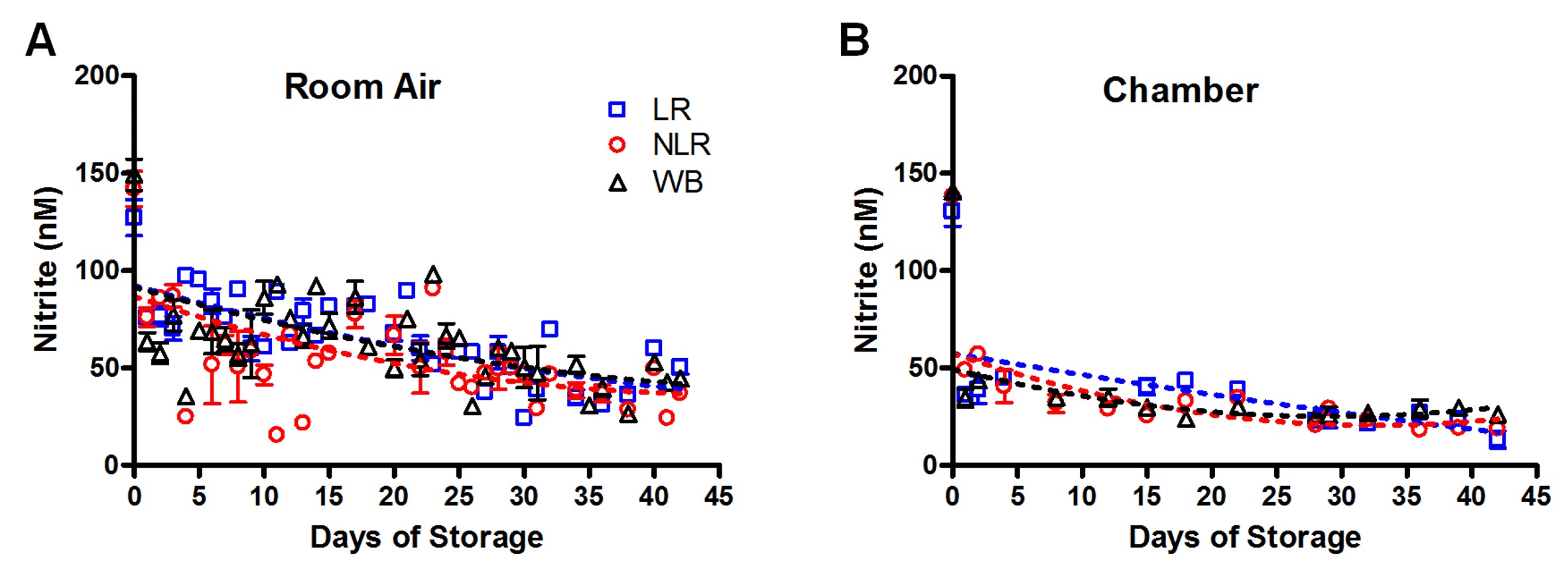

Supplement: Changes in the three blood forms nitrite levels over the duration of storage. — Figure 2. Time-dependent changes in nitrite concentration during storage. Blood components stored in the three forms noted were kept for 42 days at 4°C in either room air (2A) or an argon chamber (2B), to emulate aerobic and hypoxic conditions, respectively; number of donors, n=3 (A), n=3 (B). Supplemental Figure 2. Time-dependent room air and chamber changes in nitrite concentration for individual stored blood components; number of donors, n=3 (room air), n=3 (chamber). To view the data behind the graphs, access 'show all items' above. [file f1000research-1-212-s0000.tgz › figure2.jpg]

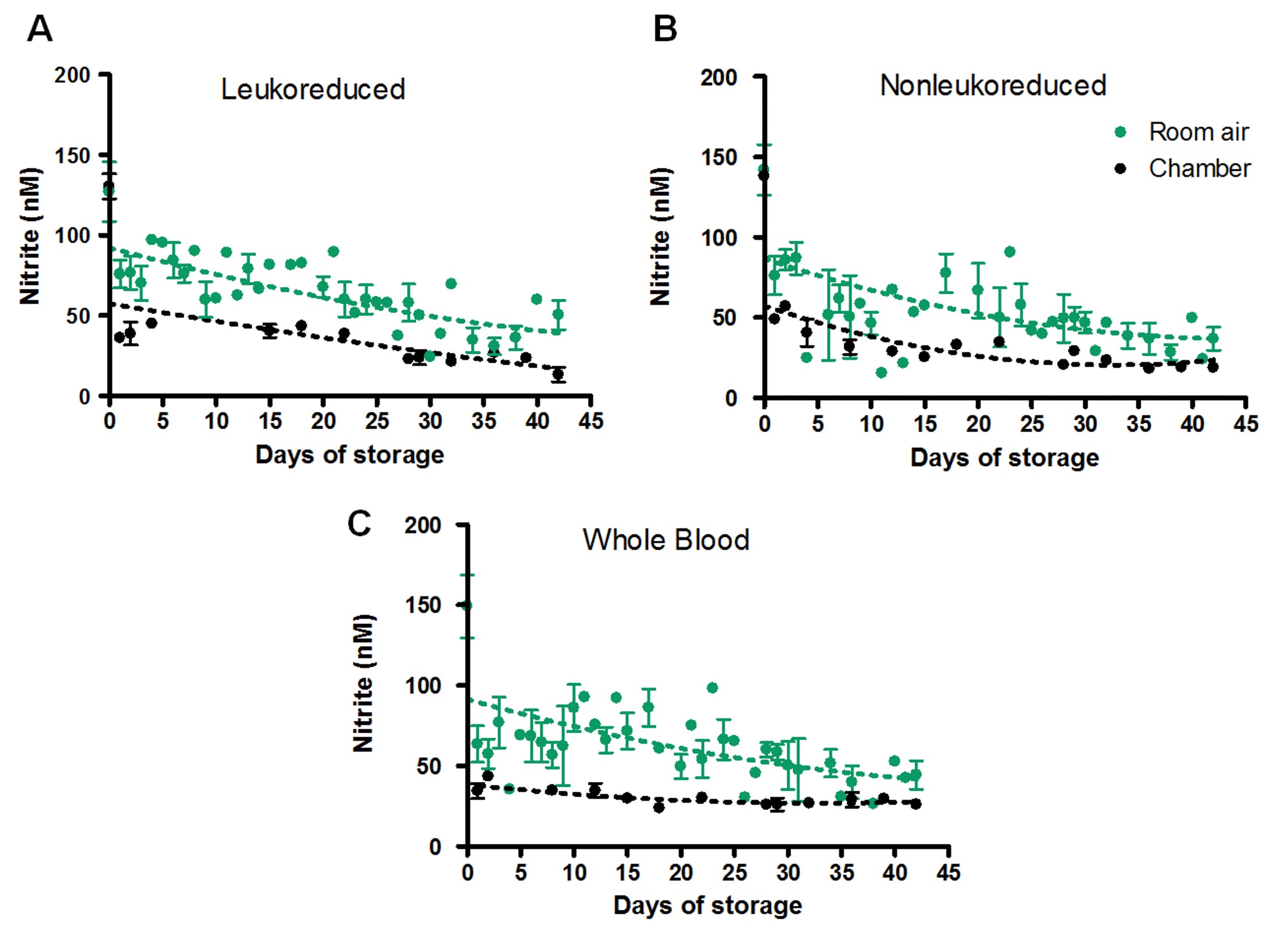

Supplement: Changes in the three blood forms nitrite levels over the duration of storage. — Figure 2. Time-dependent changes in nitrite concentration during storage. Blood components stored in the three forms noted were kept for 42 days at 4°C in either room air (2A) or an argon chamber (2B), to emulate aerobic and hypoxic conditions, respectively; number of donors, n=3 (A), n=3 (B). Supplemental Figure 2. Time-dependent room air and chamber changes in nitrite concentration for individual stored blood components; number of donors, n=3 (room air), n=3 (chamber). To view the data behind the graphs, access 'show all items' above. [file f1000research-1-212-s0000.tgz › supplemental_figure2.jpg]

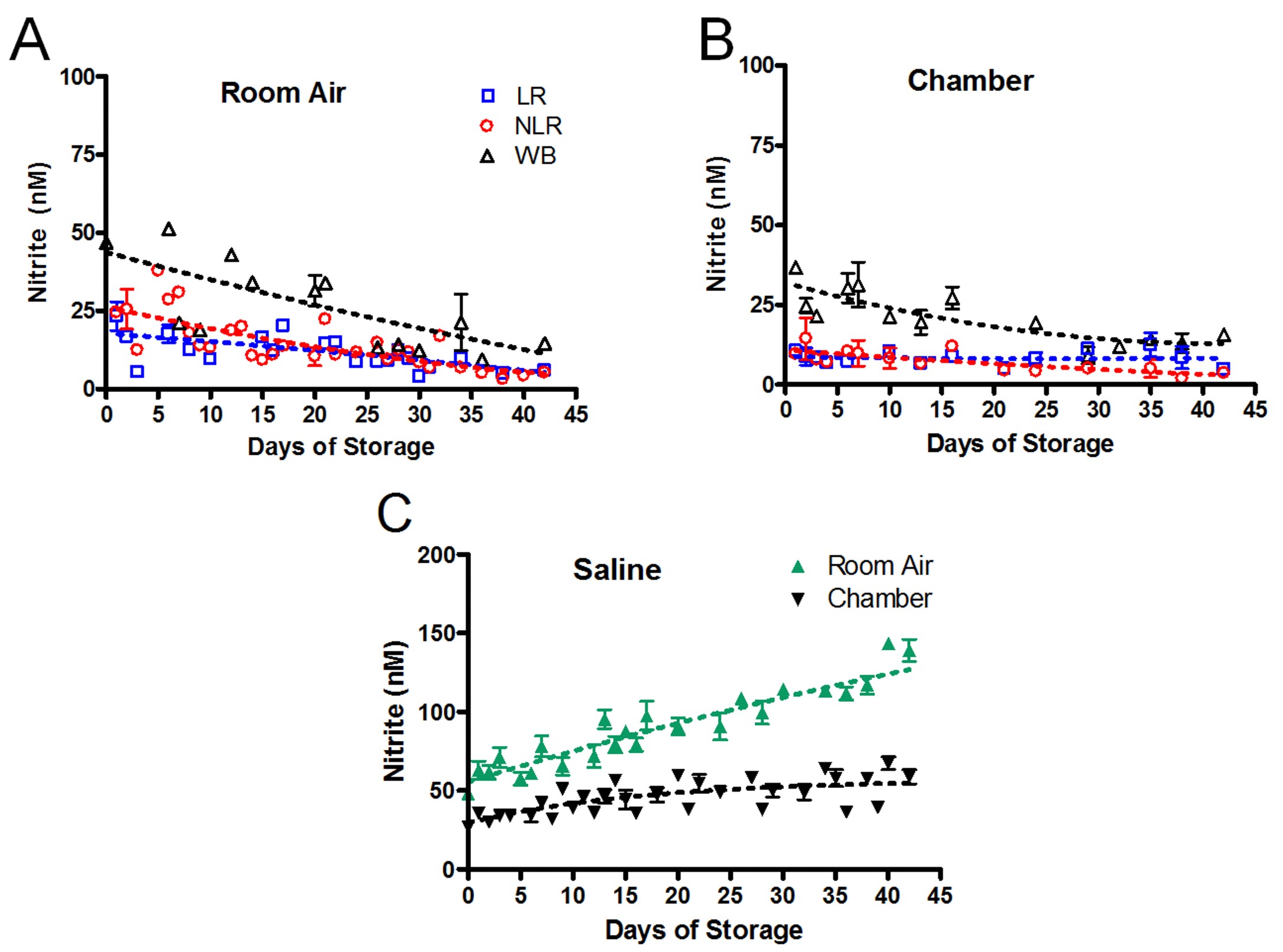

Supplement: Nitrite concentration in supernatants and saline stored in room air and argon chamber. — Nitrite concentration in supernatants and saline stored in room air or an argon chamber. Fig. 3A shows the nitrite concentration in supernatants stored in room air, number of donors, n=3, while Fig. 3B shows the same for supernatants stored in an argon chamber, number of donors, n=3. Nitrite concentrations in saline controls stored under both conditions are shown in Fig. 3C, number of donors, n=6 (room air n=3, argon chamber n=3). To view the data behind the graph, access 'show all items' above. [file f1000research-1-212-s0001.tgz › figure3.jpg]

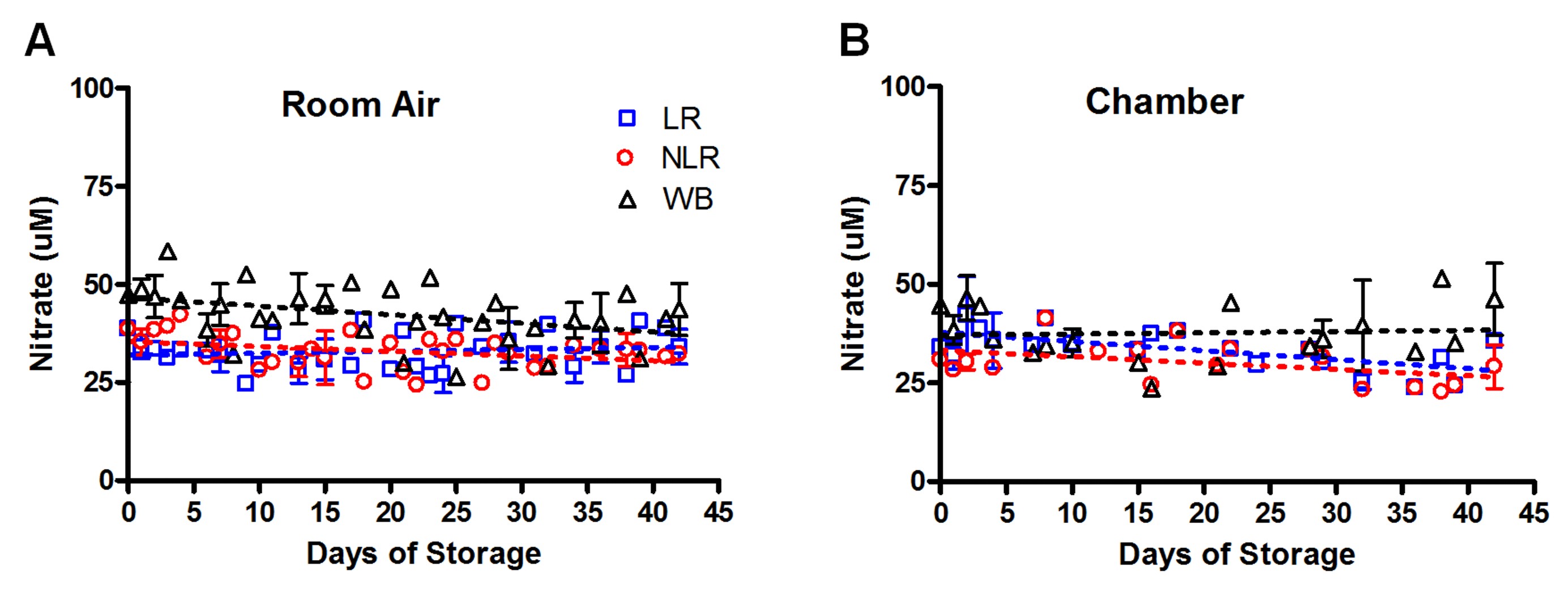

Supplement: Effect of storage on the three blood forms nitrate concentration — Effect of storage on nitrate concentration in blood stored for 42 days in room air (4A) or an argon chamber (4B); number of donors, n=3 (A), n=3 (B). To view the data behind the graphs, access 'show all items' above. [file f1000research-1-212-s0002.tgz › figure4.jpg]

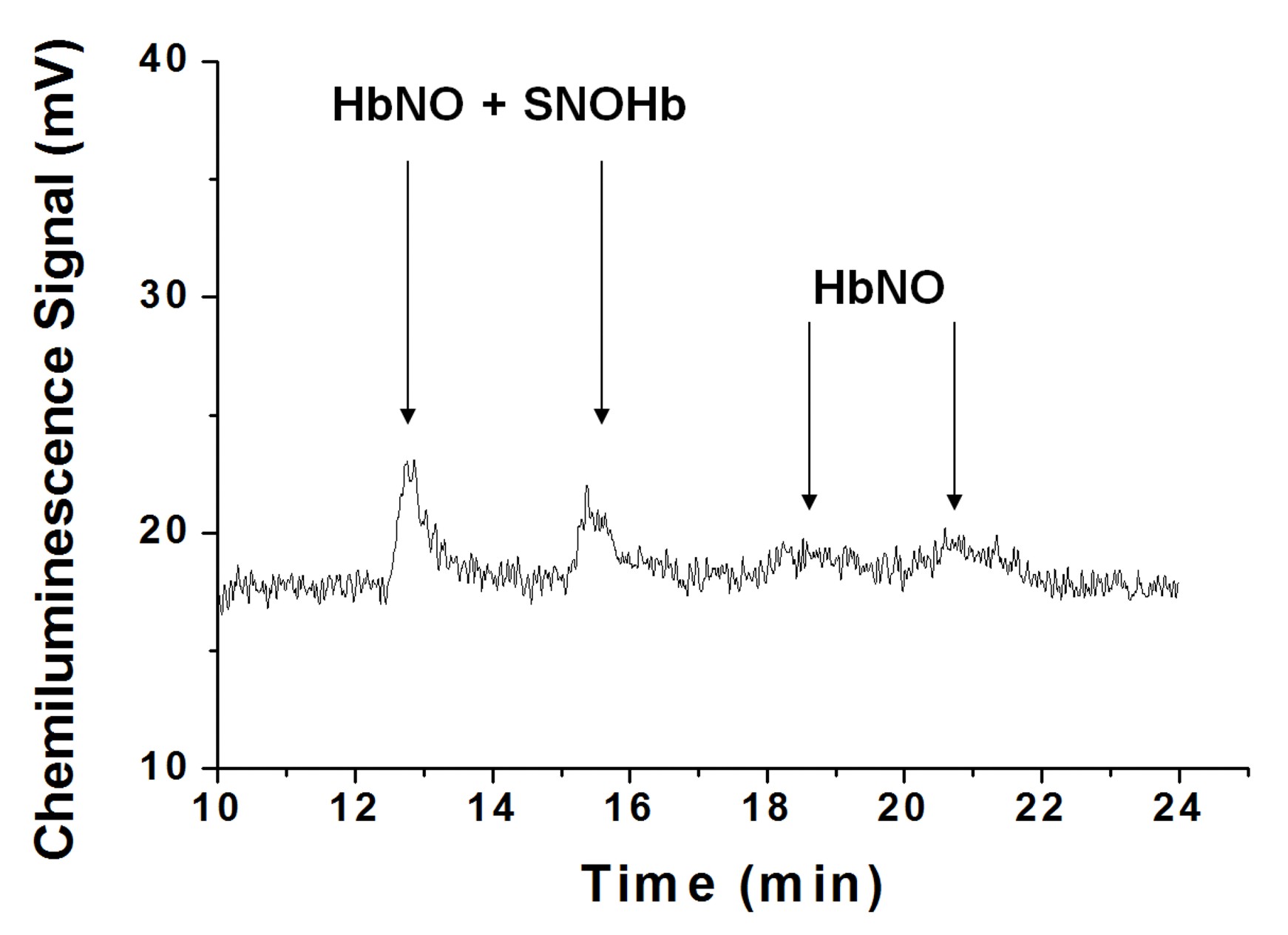

Supplement: HbNO and SNOHb levels — SNOHb levels in fresh blood (assay performed in the first hour after venisection). Gas-phase chemiluminescence signals used to determine SNOHb concentration. The peaks from two samples in the first 20 minutes of storage are shown; SNOHb concentration is ascertained by subtracting the HbNO peak from the composite of SNOHb plus HbNO after treatment with HgCl2 and acid sulfanilamide. The values of SNOHb, near the sensitivity of the method, are less than 30nM, while HbNO is barely detectable. Neither peak was detected after 1hr of storage. To view the data behind the graphs, access 'show all items' above. [file f1000research-1-212-s0003.tgz › figure5.jpg]

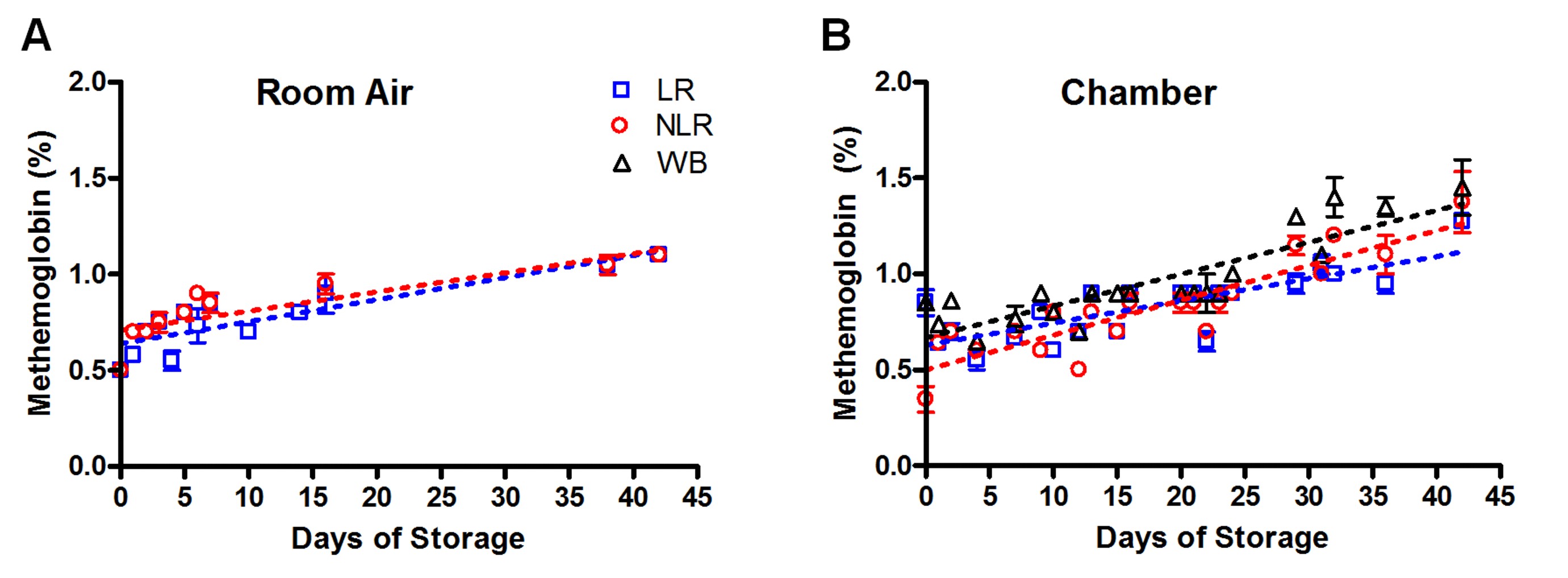

Supplement: MetHb levels in room air and argon chamber. — Change in MetHb levels in room air (6A) and chamber (6B) samples over 42 days of storage; number of donors, n=2 (A), n=2 (B). To view the data behind the graphs, access 'show all items' above. [file f1000research-1-212-s0004.tgz › figure6.jpg]

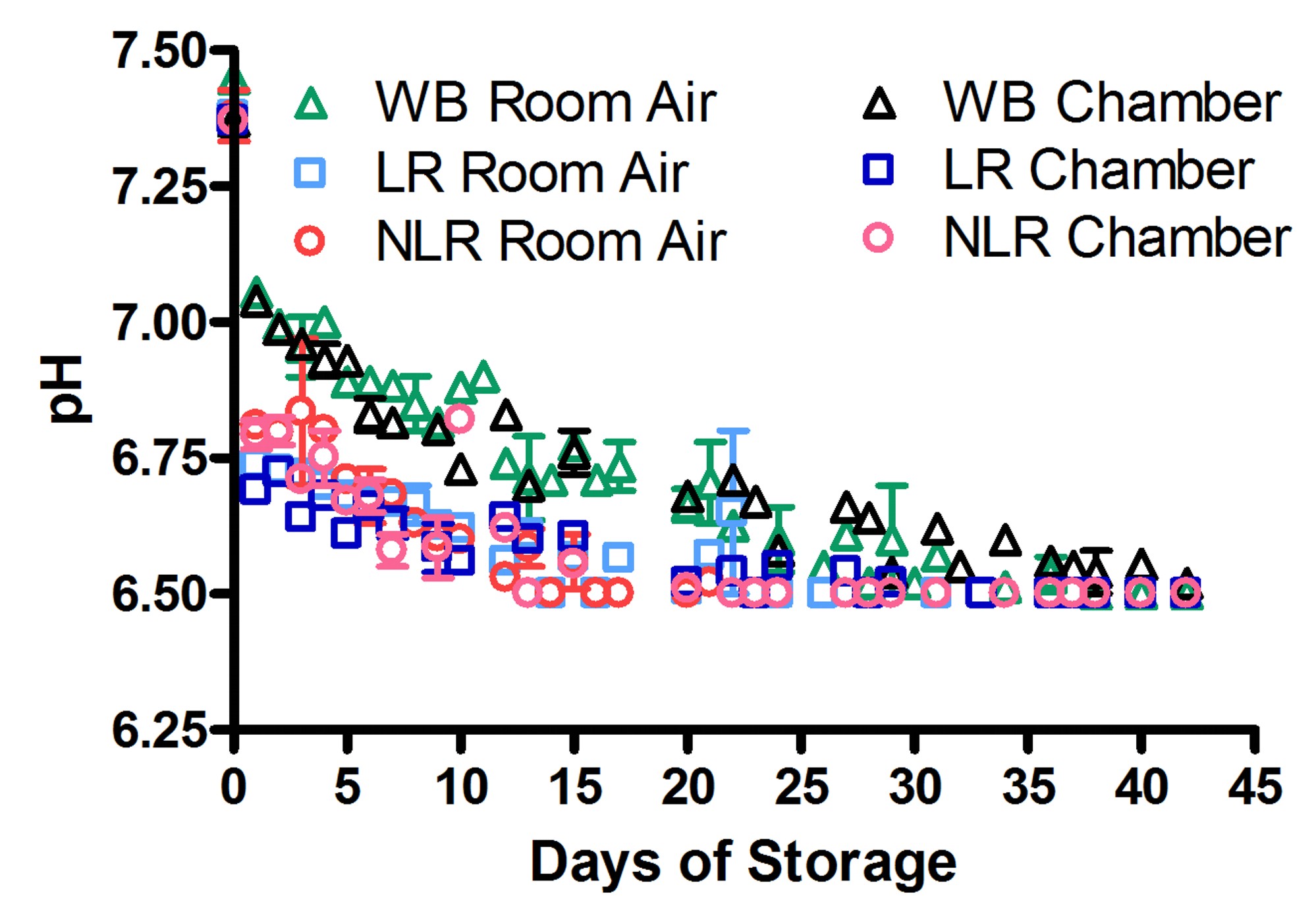

Supplement: Room air and argon chamber comparisons of pH levels in stored blood. — Room air and argon chamber comparisons of pH levels in stored blood; number of donors, n=3 (room air), n=3 (chamber). Note: i-STAT apparatus does not detect pH levels below 6.5; values read as < 6.5 were plotted at 6.5. To view the data behind the graph, access 'show all items' above. [file f1000research-1-212-s0005.tgz › supplemental_figure3.jpg]

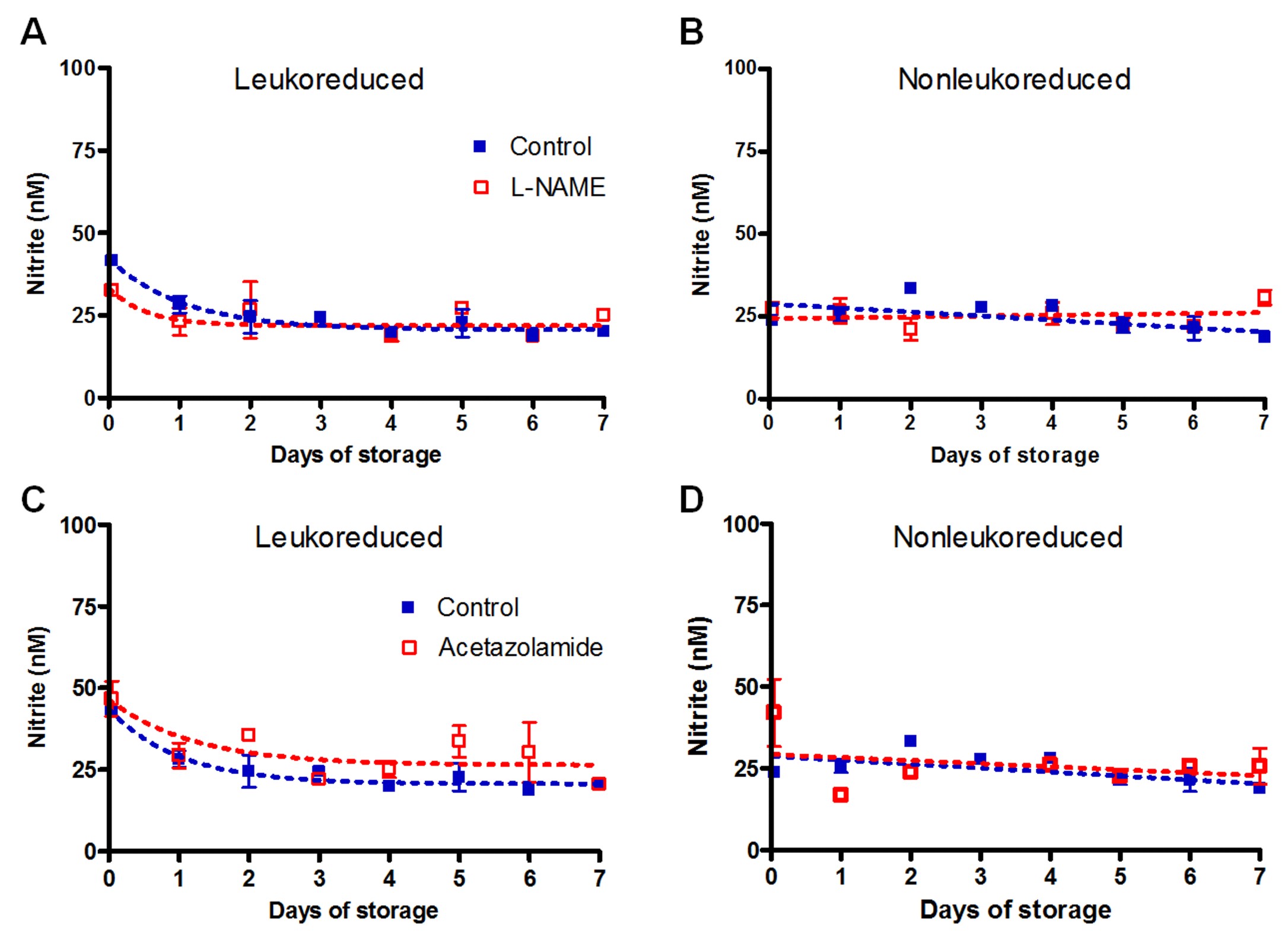

Supplement: Inhibition of NO-producing enzymes — Change in MetHb levels in room air (6A) and chamber (6B) samples over 42 days of storage; number of donors, n=2 (A), n=2 (B). To view the data behind the graphs, access 'show all items' above. [file f1000research-1-212-s0006.tgz › supplemental_figure4.jpg]
