# Supplementary material for: Effect of storage levels of nitric oxide derivatives in blood components
Source: F1000Res. 2012 Oct 22;1:35. [Version 1] doi: 10.12688/f1000research.1-35.v1 (PMC3814924; doi:10.12688/f1000research.1-35.v1)
Supplement: Peer review history

# Peer review: Referee response for version 1

**Published:** 2012-11-12

**Authors:** Jay Zweier

**Affiliations:** 1 Davis Heart & Lung Research Institute, The Ohio State University, Columbus, OH, USA

Overall this research article is well written and clear for the most part. I do however have 2 minor comments for the authors to think about:

1. It would be interesting to include data on the time course and extent of hemolysis as this may contribute to toxicity and also the nitrite depletion.

2. The presentation of Figure 5 is unclear. The authors should better describe and explain this figure. 2 sets of 2 peaks are seen at different times. Please explain the times shown for each peak. Please explain each peak and exactly what it is and how it was measured. It is noted that 2 samples are shown, which correspond to which peak? What is the difference between these 2 samples? How does this relate to the time shown? Were there quantitative standards that were run for HbNO and SNOHb? Could some portion of these small peaks arise from residual nitrite?

I have read this submission. I believe that I have an appropriate level of expertise to confirm that it is of an acceptable scientific standard.

# Response

**Published:** 2012-11-19

**Authors:** Barbara Piknova

**Affiliations:** Molecular Medicine Branch, National Institutes of Diabetes and Digestive and Kidney Diseases, National Institutes of Health, USA

We thank Dr Zweier for his approval and comments.

**1.** It is true that hemoglobin from RBCs lyzed during the storage over 42 day would significantly alter the amount of nitrite measured in preparation, as hemoglobin is a very potent nitrite oxidase. However, during our study we did not observe any significant hemolysis in our preparations (in fact, using spectrophotometry, Hb was below our detection limits in RBC supernatants) and data from other studies from NIH blood bank were also in favor of only minor hemolysis occurring during the careful handling of stored blood.

**2.** Figure 5 is an example of our raw chemiluminescence data i.e. the output from the NOA analyzer. The principle and application of this method is described in details in Piknova B and Schechter AN (2011) Measurement of nitrite in blood samples using the ferricyanide-based hemoglobin oxidation assay. Methods Mol Biol. 704:39-56.

In this method, reaction vessel is filled with nitrite/HbNO/SNOHb-reducing tri-iodide solution and connected to NOA analyzer. Carrier gas (He) passing through the reaction solution carries NO from reduced “NOx-compounds” into reaction chamber inside the NOA analyzer, where NO reacts with ozone (made by high-voltage generator from oxygen) and forms NO*. When excited NO* molecule returns to its main electron levels, a photon is emitted in red-light region and registered by photomultiplier. The reaction ratio is 1:1 in NO:NO* and also in NO*:photon, which allows to very precise quantification of NO and therefore, original nitrite/HbNO/SNOHb species present in sample. The specificity towards HbNO and SNOHb is achieved by different pre-treatments of sample and by subtraction of known components from composite peaks. In Figure 5 we tried to illustrate that peaks obtained for SNOHb+HbNO were already somehow on the detection limit of our method and that trying to quantify them would lead to significant errors. Therefore, we did not proceed to quantification of these compounds, as we know from our previous experiments that levels comparable with Figure 5 would be from ~20nM HbNO and SNOHb, which was really the low detection limit. Times shown on Figure 5 are for illustrative purposes only, subsequent injections of different samples into vessel are possible whenever the previous sample readings returns to the baseline.

# Peer review: Referee response for version 1

**Published:** 2012-10-29

**Authors:** Malte Kelm

**Affiliations:** 1 Division of Cardiology, Pulmonology, and Vascular Medicine Medical Faculty, University Duesseldorf, Duesseldorf, Germany

In the present study, Qazi *et al*. examined the effect of storage on levels of nitric oxide derivatives in blood components.

They examined the biochemical changes in the nitric oxide metabolism in red blood cell transfusions and reported that nitrite concentrations initially decreased rapidly and stabilized afterwards for up to 42 days. Under hypoxic conditions in an argon chamber there are even less nitrite levels compared to room air.

In my opinion the data provided by the authors is interesting as they show a long-term view on nitrite levels in red blood cell transfusions and provide valuable information about optimizing the preservation of stored blood. These findings definitely extend the current knowledge base in the field.

I have read this submission. I believe that I have an appropriate level of expertise to confirm that it is of an acceptable scientific standard.

# Peer review: Referee response for version 1

**Published:** 2012-10-25

**Authors:** Eddie Weitzberg

**Affiliations:** 1 Department of Physiology and Pharmacology, Karolinska Institutet and Hospital, Stockholm, Sweden

I have read this submission. I believe that I have an appropriate level of expertise to confirm that it is of an acceptable scientific standard.

# Peer review: Referee response for version 1

**Published:** 2012-10-24

**Authors:** Jon Lundberg

**Affiliations:** 1 Department of Physiology and Pharmacology, Karolinska Institutet and Hospital, Stockholm, Sweden

I have read this submission. I believe that I have an appropriate level of expertise to confirm that it is of an acceptable scientific standard.

# Peer review: Referee response for version 1

**Published:** 2012-10-23

**Authors:** Andre Dejam

**Affiliations:** 1 Department of Internal Medicine, Brigham and Women's Hospital, Boston, MA, USA

I read with great interest the article by Dr. Schechter’s group in DC on the effect of storage on levels of nitric oxide derivatives in blood components.

I approve this article as it extends the current knowledge base in the field.

I have read this submission. I believe that I have an appropriate level of expertise to confirm that it is of an acceptable scientific standard.
